# Predictors of emphysema progression in HIV-positive patients

**DOI:** 10.7448/IAS.17.4.19660

**Published:** 2014-11-02

**Authors:** Giovanni Guaraldi, Antonella Santoro, Giulia Besutti, Riccardo Scaglioni, Guido Ligabue, Stefano Zona, Paul Man, Don Sin, Jonathon Leipsic, Cristina Mussini

**Affiliations:** 1 Infectious Disease, Policlinic Hospital, University of Modena and Reggio Emilia, Modena, Italy; 2 Radiology, Policlinic Hospital, University of Modena and Reggio Emilia, Modena, Italy; 3 Respiratory Medicine, Saint Paul Hospital, British Columbia University, Vancouver, Canada; 4 Radiology, Saint Paul Hospital, British Columbia University, Vancouver, Canada

## Abstract

**Introduction:**

The aim of the study was to find factors associated with emphysema progression (EP), assessed on sequential thoracic CT scans, in a large cohort of HIV-positive patients.

**Materials and Methods:**

This was an observational, prospective study of 448 consecutive HIV-positive patients on antiretroviral therapy who underwent two sequential ECG-gated coronary artery calcium scoring CT scans. Images were reviewed by three radiologists by consensus to assess lung emphysema using a visual semi-quantitative score (0 to 4) for each of six lobes. EP was defined as an increase in emphysema score. Heavy smoking habit was defined as a self-reported number of cigarettes per day smoked greater than 10. Immune reconstitution was defined as the change in CD4 cell count between first CT scan and CD4 nadir and it was divided into tertiles. Progressors and non-progressors were compared using X2-test for categorical variables and T-test of Mann–Whitney U test for continuous variables where appropriate. Factors independently associated with EP were explored using multivariable logistic regression analyses. A p-value <.05 was considered statistically significant.

**Results:**

The mean age of the included patients was 47,9±7,7 years, 24,1% of them were females and 39,3% were smokers. The median interval between the two CT scans was 2,4 years (interquartile range 0,69–5,9 years). EP was significantly associated with HIV-infection duration (p=0,056), smoking (p=0,007) and in particular heavy smoking habit (p=0,015) and time interval between the two scans (p=0,021), while the highest tertile of immune reconstitution was borderline in significance (p=0,075). Age and sex were not significantly related to EP and were not included in further analyses. HIV infection duration (OR=1,01; p=0,013), time interval between the two scans (OR=1,51; p=0,032) and heavy smoking habit (OR=3,36; p=0,041) remained independently associated with EP in multivariate analysis.

**Conclusions:**

In this large cohort of HIV positive patients on antiretroviral therapy, HIV infection duration, time between CT scans and continued heavy cigarette smoking were independently associated with EP.
